# Experimental philosophy of medicine and the concepts of health and disease

**DOI:** 10.1007/s11017-021-09550-3

**Published:** 2021-12-01

**Authors:** Walter Veit

**Affiliations:** Charles Perkins Centre, University of Sydney, Sydney, Australia

**Keywords:** Philosophy of medicine, Experimental philosophy, Conceptual analysis, Health and disease, Boorse, Naturalism

## Abstract

If one had to identify the biggest change within the philosophical tradition in the twenty-first century, it would certainly be the rapid rise of experimental philosophy to address differences in intuitions about concepts. It is, therefore, surprising that the philosophy of medicine has so far not drawn on the tools of experimental philosophy in the context of a particular conceptual debate that has overshadowed all others in the field: the long-standing dispute between so-called naturalists and normativists about the concepts of health and disease. In this paper, I defend and advocate the use of empirical methods to inform and advance this and other debates within the philosophy of medicine.

*There is a widespread and unfortunate tradition in philosophy that the man in the street has all the empirical knowledge required for philosophizing.*

*– Daniel C. Dennett [* 1 *, p. 1]*

## Introduction

If one had to identify the biggest change within the philosophical tradition in the twenty-first century, it would certainly be the rapid rise of experimental philosophy. What began as a small initiative to promote empirical methods within philosophy and test the intuitions of so-called armchair philosophers has led to a wealth of studies on the intuitions of the public concerning a diverse range of philosophical subjects in epistemology, ethics, metaphysics, and even aesthetics. The field has quickly grown into a lively community but faces criticisms from both inside and outside the discipline of philosophy.

Owing to an array of defences by respected philosophers of science, experimental methods have become increasingly common and accepted within the philosophy of science (see [2–6]). While this has led to major changes in thinking and a more pluralist attitude towards the use of scientific tools within philosophy of science itself, it would be premature to call this a victory for experimental philosophy of science. Indeed, there is one subdiscipline of philosophy of science in which experimental philosophy is notably absent: the philosophy of medicine. I think this is unfortunate and would like to use this opportunity to remedy this omission.

Indeed, the lack of empirical methods in the philosophy of medicine is all the more surprising given that experimental philosophy has predominantly been used to challenge many of the more intuition-grounded conceptual debates in philosophy. Experimental work on scientific concepts includes early studies on the gene concept [7], innateness [8–10], the economists’ concept of choice [11], consciousness [12], and conceptual differences between natural and social scientists more generally [13, 14]. Yet, one particular conceptual debate has overshadowed all others in the philosophy of medicine: the long-standing dispute between so-called ‘naturalists’ and ‘normativists’ about the concepts of health and disease—specifically, whether these terms refer to value-free scientific concepts or value-laden social ones. But as the quotation from Daniel Dennett in the epigraph of this paper is meant to illustrate, the very notion that a theory of health and disease could be developed by reference to one person’s intuition alone strikes me as quite strange if not absurd, and yet this seems to be the dominant view in the philosophy of medicine with mere intuitions of different philosophers being traded. This is where experimental philosophy has a useful role to play by gathering empirical data about the intuitions of a much broader class of people. In this paper, I shall defend and advocate the use of empirical methods to inform and advance the conceptual discussion of health and disease and other debates within the philosophy of medicine, a discipline that has so far resisted the empirical turn within philosophy.

The paper is structured as follows: It begins in the next section by offering a brief sketch of the philosophical literature on health and disease and articulates a problem for traditional conceptual analysis that I dub the intrinsic limitation problem, following Maël Lemoine [15]. Then the methods of experimental philosophy are introduced as a necessary solution to this problem and several of the objections philosophers of medicine are likely to raise are addressed before concluding with a discussion.

### Conceptual analysis of health and disease

The central question within the philosophy of medicine is how to define and understand concepts such as health, disease, and pathology. This philosophical question has garnered much attention throughout recent decades and continues to be one of the most heatedly debated issues in the field (see [16] for an overview). Traditionally, the contenders within this debate have been grouped into two opposing camps: *naturalists*, who try to ground the concepts in objective biological facts, and *normativists*, who argue that these concepts cannot be grounded purely in objective facts about science because they are ultimately value-laden (depending on the human viewpoint) or culturally relative.^1^ While this characterisation offers only a simplified and coarse-grained picture of the multifaceted debate on these concepts, it will be sufficient for my present purposes, since I simply aim to show that the traditional philosophical method of trying to resolve this debate—conceptual analysis—is likely to remain unsuccessful. I am not making any claims regarding the sensibility of the dichotomy in terms of which the debate has been historically framed. Whether one should be an eliminativist, pluralist, or unificationist about the conflict between normativism and naturalism in this debate is, as I shall argue, to a non-negligible extent an empirical matter. Here it is useful to take Lemoine’s recent critique of conceptual analysis as a starting point.

### The intrinsic limitation of conceptual analysis

Traditionally, conceptual analysis, or the descriptive analysis of concepts, has been assumed to play the central—if not the only—role in settling the conflict between naturalism and normativism on the meaning of health and disease. The two most influential naturalist accounts in this debate, Christopher Boorse’s bio-statistical theory and Jerome Wakefield’s harmful dysfunction analysis, have made explicit reference to conceptual analysis in their methods [19–23]. Boorse, for instance, states in his 1997 essay: ‘twenty years ago, in four papers, I offered a unified descriptive analysis of health, disease, and function’ [21, p. 4]. Historically, most of the contributors to the discussion have paid little if any attention to the meta-philosophical question of whether this method is an appropriate one. As Lemoine points out, ‘all participants implicitly agree about the utility of conceptual analysis to settle the debate’ on how disease should be defined [15, p. 316].^2^ There are a few notable exceptions, such as John Matthewson and Paul Griffiths on the naturalist side and Quill Kukla on the normativist side, who do not try to capture the meaning of the terms as used by the public but specifically try to revise the concepts of health and disease for the purposes of science and justice, respectively [25–29]. Most of what has been published in this debate, however, squarely falls under the rubric of conceptual analysis.

Since I am interested in the intrinsic limitation problem identified by Lemoine [15], according to which conceptual analysis cannot rule out stipulations about a term’s internal content, I will follow his formalisation of this methodology in order to make the problem visible.^3^ When philosophers are interested in the conceptual analysis of a term—be it ‘health’, ‘disease’, ‘pathology’, ‘disability’, or ‘illness’—they start from something cognitive scientists have called exemplar theory.

Exemplar theory postulates that humans learn and apply concepts by comparing new stimuli to stored collections of particular instances, such that a new stimulus is classified as a member of a given category if it bears a strong enough context-dependent similarity to the sum of the remembered instances, or exemplars, contained within that category [30]. While this strategy of intuitive categorisation might be enough in common practice to categorize pathologies such as an infection, it is unsatisfying to most philosophers of medicine who want a *theory* of health and disase.^4^ Instead of simply comparing new cases against a set of uncontroversial cases of health or disease, philosophers aim to construct a definition from the latter. This definition must provide the necessary and sufficient conditions by which a case can be classified as healthy or pathological and may include exceptions to these criteria in order to provide against easily constructed counterexamples [15, p. 310]. With a successful definition of health or disease in place, philosophers should be able to place controversial cases either inside or outside the set of instances designated by the term (called its *extension*). Progress, it is assumed, proceeds by subjecting these definitions to attacks that would warrant their revision or replacement in hopes of coming closer to the correct account of health and disease. Lemoine identifies three distinct kinds of attack to that effect:

(1) cases falling within the commonly accepted extension of the term but which do not satisfy the opponent’s definition, (2) cases that do satisfy the opponent’s definition but which fall outside the commonly accepted extension, and (3) cases that fall clearly inside or outside the extension but which the opponent’s definition fails to classify at all. [15, p. 310]

Boorse characterises this back-and-forth process of definition and attack as a game: ‘to call pregnancy per se unhealthy would strike at the very heart of medical thought; it is the analytic equivalent of the “Game Over” sign in a video game’ [21, p. 44]. And this game sure has had many contenders. Despite being doubtless the most discussed work in the literature, Boorse’s decades-old bio-statistical theory—first introduced in 1977 [20]—is far from well accepted. While there have been slight amendments to Boorse’s original account, the continual assault against it in the literature is hardly indicative of a process apt to culminate in the establishment of a single prevailing definition: there are now more accounts rather than less. One might thus question, then, whether the participants in this discussion are actually following the supposed rules of conceptual analysis, according to which one wins the debate by ‘eliminating the other contenders’ definitions and not having one’s definition eliminated’ [15, p. 311].

Nonetheless, as Lemoine shows, these rules seem to play a core role in the work of Boorse, Nordenfelt, and Wakefield; all of them ‘(1) propose a definition of health, disease, or both, (2) give examples of actual diseases, (3) examine apparent counter-examples, and (4) offer counter-examples to the contending proposals for definitions’ [15, p. 311]. Definitions are often argued to be either too narrow, excluding conditions considered to be diseases (such as septicaemia, asthma, or atherosclerosis), or too broad, including conditions that would preferably not be labelled as diseases (as in the pregnancy example above). Lemoine observes that for this kind of analysis to eventually lead to success, or at least make progress, there needs to be some manner of consensus among participants in the debate as to which conditions count as healthy or pathological. But the need for such consensus extends only to the clear cases a concept is intended to capture—since controversial cases can easily be dismissed as unclear out of hand, it is the inclusion of consensually healthy conditions or exclusion of consensually diseased conditions that is damning for a definition of disease. In that way, the argumentative process of conceptual analysis will not lead to a definition that rules controversial cases in or out. As Lemoine points out, ‘if several definitions could match the same set of uncontroversial cases, it would not matter whether they agreed or disagreed on controversial cases [15, p. 316]. Insofar as multiple different definitions of a concept are left standing in the face of uncontroversial counterexamples, then, conceptual analysis alone will not be able to settle the game and pronounce a winner. This is an ‘intrinsic limit of conceptual analysis’: it cannot rule out specific claims about how a concept should be defined [15, p. 316].

Whether the solution to the conceptual controversy about health and disease is found in the naturalist or normativist camp cannot be established using conceptual analysis if both sides can offer accounts that cover the uncontroversial cases equally well. I see no a priori reason to doubt that there is conceptual room on both sides for such definitions. The debate is thus, as Lemoine argues, ‘hopelessly unlikely to decide between two reasonably successful definitions of “disease” or “health”’ [15, p. 323]—that is, if its participants limit themselves to conceptual analysis alone.^5^ Lemoine concludes his paper with the declaration that ‘in order to prove naturalism or normativism right, another method has to be embraced’ [15, p. 324]. I shall follow this call for action and introduce another method in the philosophy of medicine that can make empirically supported judgments regarding intuitions about and uses of concepts in the public domain and medical profession—namely, experimental philosophy (sometimes abbreviated as x-phi or XPhi).^6^

### Experimental philosophy to the rescue

That Lemoine’s call for an alternative method can be used to offer a defence of experimental philosophy of medicine comes as no surprise. The criticisms Lemoine brings forward against conceptual analysis have been echoed in similar form by many naturalist philosophers, and especially those that advocate the use of empirical methods. Experimental philosophers (among others) have long criticised the tendency of philosophers to rely on their own intuitions to posit the meaning of a term, rather than the intuitions of the general population whose usage they are supposedly capturing [36]. Here one might legitimately wonder whether intuitions—be they philosophical, scientific, or lay—are actually helpful to understand the target of a concept. Why should it be assumed that mere intuitions can help settle these conceptual questions, especially those of broader (non-specialist) populations?

Unfortunately, experimental philosophy is often narrowly understood as the use of surveys to capture the intuitions of the public, rather than as a more general view of philosophy as a discipline continuous with the sciences and thus apt to use empirical data to inform its debates. A naturalist understanding of the x-phi critique of conceptual analysis turns the usual critique of x-phi on its head. Rather than criticising the use of empirical methods to address philosophical issues as something that is not philosophy, a naturalist criticises philosophical debates for a lack of empirical tools. In promoting x-phi, I aim to spark an empirical turn in the philosophy of medicine similar to the one initiated by Dennett in the philosophy of mind. In his words:

As a graduate student at Oxford (1963-65), I developed a deep distrust of the methods I saw other philosophers employing. That was the heyday of ordinary language philosophy, and ‘theories of mind’ were debated on the basis of a lean diet of conceptual analysis—as if one could develop a theory of horses on the basis of nothing other than a careful investigation of the meaning of the ordinary word ‘horse’. I decided that I had to supplement (and maybe even adjust!) the fruits of ordinary language analysis with an attempt to figure out how the brain could possibly accomplish the mind’s work. [37]

What I am advancing here is not merely the uncontroversial claim that empirical methods can improve debates in the philosophy of medicine, but also the more substantial one that empirical methods are necessary to make progress in the general conception of health, disease, and pathology. Understanding x-phi as part of the larger project of naturalist philosophy makes it naturally fit with two distinct naturalist critiques of conceptual analysis that are often brought forward against x-phi: (i) concepts are not definitions and (ii) intuitions are a poor guide to understanding natural phenomena. First, x-phi may very well reveal that the way people categorise the world cannot be captured in simple definitions made up of necessary and sufficient conditions. But this finding would go to show only that conceptual analysis has rested on mistaken assumptions, not that x-phi has somehow failed because it cannot provide such conditions. Second, naturalists should be happy to make a distinction between people’s psychological categorisations and the way the world is. Conceptual analysis is sometimes ambiguously understood either as the search for a concept’s essence or true meaning or as a mere set of ‘criteria of application’ that actual humans use when employing certain concepts [38, p. 171], but these goals are distinct. Naturalists can both engage in a scientific investigation of how people end up with and employ concepts in communication with others and their own thinking and engage in a scientific investigation of whether these concepts map onto real phenomena in nature. Whereas Lemoine, along with others such as John Matthewson and Paul Griffiths [25], abandons conceptual analysis in favour of the latter approach—that is, grounding concepts in science [15, 39]—this paper will advocate the former approach, using surveys of the public, medical practitioners, and scientists to advance the debate between so-called naturalists and normativists. Importantly, however, these approaches are not incompatible and can inform each other, especially where scientists’ intuitions diverge from their empirical findings, as Griffiths and colleagues’ work on the idea of innateness and the gene concept elegantly illustrates [7–10]. The once purely philosophical project of conceptual analysis is thus replaced by two distinct investigations: one an empirical investigation of the psychology and sociology behind our concepts, the other a scientific investigation of the phenomena these concepts supposedly map onto.^7^

Now, while it is surely justifiable to ask why one should be a naturalist and rely on empirical data at all, this is not the place to answer these concerns. This is a paper about the philosophy of medicine, a field where scientific approaches such as those of Lemoine, Griffiths, and Matthewson are rare and x-phi is notably absent. Due to considerations of space, I give only a positive account, showing how experimental philosophy could be used to improve the philosophy of medicine by discussing its most famous conflict. If this approach is meant to fail, its failure will likewise have to be empirically demonstrated and not a priori asserted.

### Conceptual analysis requires experimental philosophy

The primary argument for experimental—or, better yet, *empirical*—philosophy of medicine is this: successful conceptual analysis in its nature requires empirical data. Here a lexicographical approach is illustrative and shares a close connection to traditional conceptual analysis.

In the course of compiling a lexicon, lexicographers will select a list of examples representative of a word’s usage and then either organize these examples into different senses or present a definition that covers all uses of the word. However, a single definition is often not provided or may be stated in quite inclusive terms so as to not exclude any examples. One reason for this is simply pragmatic: some lexical items are applied in very different contexts in which they have different meanings that cannot be unified under a single concept. The concepts of health and disease are taken to be radically different in that regard, assuming that there is a single ‘correct’ unifying concept. If the goal is conceptual analysis of a term as used by a linguistic community, one cannot take one’s own intuitions as a starting point, unless there is evidence that these intuitions are widely shared. Indeed, it is doubtful that one could even engage in such conceptual analysis without empirical methods. Irrespective of the tools philosophers use to get a grasp on these shared intuitions—whether they rely on interviews, surveys, bibliographic data, or participation in or observation of conversations about health and disease—even the very use of a lexicon to begin one’s conceptual analysis is empirical (though one might prefer to call this ‘light x-phi’ compared to more sophisticated and empirically demanding quantitative or qualitative studies).

### Experimental philosophy is not philosophy

With this in mind, I will turn to a frequent challenge brought against experimental philosophy—that while it may be useful, it does not qualify as philosophy. One might then argue that philosophers should minimise their use of empirical methods accordingly, insofar as engaging in them would take time away from doing ‘real’ philosophy. This challenge, however, is no obstacle to the necessity of the kind of work advocated and carried out by those within the experimental philosophy community: if empirical methods such as surveys do not count as philosophy, then neither will opening a lexicon or reading medical papers. There is no line to draw here.

The semantic dispute about whether or not this work should be called philosophy does not matter. It is work that needs to be done in order to make progress on philosophical questions. Yet sociological, psychological, or scientometric studies on the concepts of biomedical scientists or the public are relatively rare. After all, non-philosophers are generally not interested in particularly philosophical questions (with, perhaps, the exception of moral psychologists). So if good empirical evidence is necessary for conceptual analysis to be successful, and such empirical evidence is not otherwise collected, then philosophers occupied in conceptual debates may very well be forced to utilise the toolkit of science—that is, if they are ultimately interested in answering their philosophical questions and not merely engaged in a form of philosophical play.

But how can experimental philosophy be used to address the intrinsic limitation problem of conceptual analysis identified by Lemoine? As mentioned above, at the core of any conceptual analysis of disease is a set of uncontroversial paradigm cases that any proposed definition needs to be able to cover and plausibly also a set of cases that it ought to exclude such as pregnancy. The present state of the debate suggests that the current set is too small to allow for a determinate winner or even to make substantial progress. Empirical methods like surveys can be used to widen the set of phenomena that must be accounted for under a given definition, particularly in talk of pathology within the biomedical sciences which may provide further paradigmatic cases that should be included.

One way of widening the set would be to confront the public with controversial cases of diseases at the forefront of the conflict between normativists and naturalists. Another would be to expand the set of paradigm cases by considering the intuitions of the scientific community regarding the nature of medical conditions such as diabetes, autism, and viral infections that do not cause any felt harm but are nevertheless harmful to the organism (e.g., mosaic virus in trees). Sophisticated surveys might even attempt to discover the more abstract reasoning behind the classification process of participants. Are they drawing on scientific facts or the values of society?

Normativists may be reluctant to adopt this procedure, since the reliance on naturalistic methods could appear to unfairly shift the debate in favour of naturalism, especially when directly investigating the intuitions of scientists rather than the public at large. Expanding the set of paradigm cases to encompass any sense of pathology in science might offer the impression that the game has become rigged, in that many normativists believe that the definitions of health and disease should be based not on scientific practice but on the common understanding of these notions. This potential for rigging the game by giving preferential treatment to the folk understanding of health and pathology might be called the dice-loading problem of naturalist experimental philosophy. Yet I contend that the dice-loading problem is immaterial insofar as experimental methods need favour neither a naturalist nor a normativist account of health and disease: the ‘winner’ of the conceptual game is determined—as with anything else in experimental philosophy—empirically.^8^

There are two possible results for a study in experimental philosophy of medicine on the concepts of health and disease: either there is found to be substantial agreement about the extension of these terms among the public, medical practitioners, bioethicists, and biomedical scientists or there is found to be substantial—perhaps insurmountable—disagreement. Finding consensus that increases the number of uncontroversial paradigm cases of disease (and uncontroversial paradigm cases of non-disease states) should at least be able to foster more harmony within the conceptual literature on health and disease. Since any successful analysis would be required to cover these cases, the intrinsic limitation problem could be minimised. The use of experimental methods would create a dice-loading problem only if there were a priori reasons to think that the empirical data would inherently support one view over another. But this is not so: experimental philosophy of medicine might just as well find that there is barely any consensus on the concepts of health and disease. Finding such a lack of consensus would make the case against naturalism much stronger, favouring those normativist accounts that relativise the concept to human interests and cultural dynamics. Let me turn now to examine both possibilities—finding disagreement and finding agreement, respectively.

### Pluralism and elimination

Brian Robinson et al. and James Beebe and Finnur Dellsén have shown that scientists in different fields not only hold different methodological standards, but also interpret philosophical concepts such as objectivity and realism in very different ways [13, 14].^9^ An anecdote is useful here: the behavioural economist George Loewenstein observes that economists seem to have a much more thorough and empirically supported take on what constitutes a ‘theory’, for instance, as compared to how psychologists understand the term.^10^ Such observations are relevant to the project of experimental philosophy of medicine insofar as the use of terms that have different meanings in medical practice, paleopathology, immunological research, bioethics, evolutionary biology, and wider public use could impact on the nature of results pertaining to the concepts of health and disease. In using experimental methods to investigate whether those in different fields fall more into the normativist or naturalist camp, then, it is important to consider the nature of the questions asked. For example, if a study were to employ a simple survey-based methodology asking ‘Is health an objective measure?’ and find that the vast majority of participants across fields respond ‘yes’, this concurrence still might not reveal much about the concept of health held by the respondents, given that the concepts of objectivity and measurement are likely to vary among disciplines. The same goes for diverging results that may arise between fields: what initially appears as a difference in how, for instance, paleopathologists and medical practitioners understand the concept of health might really be an artefact of differences in how they conceive of ‘objective measures’. These difficulties are not insurmountable, but it is important to recognise them when designing appropriate questionnaires.

Further, Robinson et al. note that questions probing scientists’ intuitions may be ambiguous to the extent that it is unclear whether participants are being asked to make judgments about a concept like the gene across the sciences or within their specific field [13]. Such challenges should be taken seriously prior to experimental design, for they could seriously hinder the descriptive analysis experimental philosophers are trying to achieve. One answer to this problem is that the results would still be meaningful since it would be both the natural scientists and the social scientists who interpret the question as one about either science in general or their specific discipline. But this is only an assumption. A medical practitioner might have a conception of disease that she takes to hold generally across all disciplines, whereas a paleopathologist might have a much more restricted understanding and see talk about health and disease as discipline relative. A veterinarian working in the agricultural industry might understand animal health in a distinctive way, narrowly relating to factors that reduce livestock production.^11^ Here the usual criteria for informative scientific surveys such as sufficiently large sample sizes apply.^12^

One possible way of avoiding some of these problems is to engage in a particular form of experimental philosophy that, as Michiru Nagatsu points out [45], has become the standard within experimental philosophy of science—namely, factorial surveys, as introduced into sociology by Peter Rossi [46, 47]. These quasi-experimental survey methods employ vignettes, (i.e., concrete hypothetical descriptions) that are intended to capture participants’ implicit norms and concepts, while allowing for a sort of experimental manipulation of variables.^13^ Popularised within social psychology research, this method has been applied fruitfully to capture the diversity of conceptualisations in the biomedical sciences.

Confronting participants with descriptions of conditions in animals could present a straightforward prospective means of testing their intuitions about health and disease. The intuitions of laypeople may support either normativism or naturalism in these cases. Of course, the views of the public may differ when compared to the views of medical practitioners, veterinarians, or evolutionary biologists. If there is no conceptual divorce between animal and human health, then at least three options will appear on the table: (1) animals cannot be diseased and animal disease is just a social construction, (2) biological differences exist between human and animal disease and to analogize them is merely to engage in some sort of ‘sympathetic regression’ from human experience to other living things [48, p. 223],^14^ or (3) there is no relevant distinction between human and non-human disease and human diabetes should be understood precisely the same as canine diabetes. One pathway for navigating these options might be to confront participants with the discovery of virophages (i.e., viruses that infect other viruses) and evaluate whether the existence of virophages changes their intuitions as to whether viruses can have diseases, especially in response to statements about viruses by microbiologists such as Jean-Michel Claverie, who argues that ‘there’s no doubt this is a living organism. … The fact that it can get sick makes it more alive’ (quoted in [53, p. 677]). Examples such as these are likely to be counterintuitive to most people, even scientists not working in microbiology, undermining the idea that experimental philosophy would lead to a dice-loading problem. By adjusting and comparing examples in different samples—for instance, broken leg in humans versus broken leg in dogs versus broken wing in birds—one should be able to test whether we are just anthropomorphising animals with high degrees of similarity to us or whether disease judgments persist even in cases considered controversial in the literature (e.g., plants).

What if the analysis ultimately shows that there is no consensus? In that event, the idea of a *conceptual ecology* will be helpful in mapping out the different functional roles of varieties of concepts [2]. One result that may be encountered is a diversity of conceptualisations of disease, even within the biomedical sciences. Veterinarians, for instance, might demonstrate a different concept of animal pathology than evolutionary biologists. When experimental philosophy is used to reveal such differences in how groups of people or scientists think, the experiments can be fine-tuned to figure out the epistemic purposes a concept serves within a given group—that is, its epistemic niche. If there is a unified concept among livestock veterinarians, one can conceive of two extreme possibilities: either the concept is merely constructed for human purposes such as animal farming and slaughter, in which case classifying animals as pathological would be akin to diagnosing people with drapetomania (a disease hypothesised to explain why African slaves fled from captivity); or despite the involvement of various special interest groups and funding for research into minimising pathologies that cause a loss of yield, it is found that there is no difference in how veterinarians and biologists treat the concept of disease—both approaching it in a way that is purely objective. The possibilities are manifold.

What such an analysis would reveal, however, is that there might not be a single unified concept of health or disease. Insurance companies, politicians, medical practitioners, evolutionary biologists, veterinarians, and the public might all have different conceptions of what it means to be diseased—thereby revealing that the notion serves a variety of purposes that perhaps cannot be accomplished using a single concept. On the one hand, normativists such as Kukla are primarily interested in the roles the concepts of health and disease play for the purposes of justice [28], with some arguing that the purely naturalist conceptions of health and disease should be eliminated as mere misapplications or anthropomorphisms of these concepts. On the other hand, naturalists such as Boorse argue that it would do better to eliminate the idea that the concept of disease has any necessary connection to concerns about who deserves treatment or not [21, 22]. Some bioethicists, myself included, have even argued that the concept of disease plays no special role in deciding who deserves treatment or medical resources more generally, focusing instead on the notion of ‘enhancement’ [54–59]; that way, a normativist account of health and disease might even be abandoned.

However, as Ingo Brigandt argues in the case of the species concept, pluralism need not imply eliminativism [60]. Scientists may very well embrace pluralism and learn to live with different concepts or models of health.^15^ Elsewhere, I have argued for a functional distinction between two kinds of conceptual engineering: moral conceptual engineering and naturalist conceptual engineering—the former aimed at moral, social, or political ends and the latter undertaken for scientific purposes [70]. Whereas moral and scientific goals dovetail nicely for concepts such as well-being or welfare, it is possible that concepts such as health and disease are simply used toward disparate ends, thus warranting a conceptual division. Boorse, for instance, has argued for a distinction between the concepts of disease and illness along those lines [19]. Alternatively, one could posit a biological disease concept called pathology, while reserving the terms disease and disability for a concept geared towards resource allocation (e.g., dysfunction plus welfare loss), in a combination of naturalism and normativism à la Wakefield [23].^16^ In maintaining such distinctions, it is not clear whether normativists and naturalists should be considered winners or losers. Nonetheless, if x-phi can help to resolve the needlessly hostile dichotomy between normativists and naturalists by challenging the very idea that there is in fact a single concept to be discovered through conceptual analysis, then my case for an empirical approach to these issues would be all the better. I will now address the possibility that empirical investigation should instead reveal consensus across scientists, medical practitioners, and the public.

### Unification

Perhaps a more optimistic result of experimental philosophy of medicine would be the straightforward expansion of uncontroversial cases of diseases. If not resolving the controversy between naturalists and normativists, such a result should bring both groups closer together. There is a sense here that experimental philosophy of medicine may indeed support naturalist conceptions. If experimental methods such as surveys show broad agreement across different domains (including the biomedical ones), the extension of the concept of disease will likely expand to include animal pathologies. But as Matthewson and Griffiths argue in their defence of naturalism, some versions of normativism would entail accepting ‘conceptual divorce between human disease and pathology as a biological phenomenon’ [25, p. 451]. If naturalism can account for all these cases of animal pathologies in addition to the narrower prior paradigm set and normativism cannot, a strong case is made for naturalism. Broadening the conditions that are considered diseased within a linguistic community in this way would then imply a failure of at least some normativist accounts that have neglected to consider pathology in the nonhuman realm. An account could no longer claim to offer the superior conceptual analysis if it fails to capture paradigm cases of health and disease in animals.

Some normativists might worry that the inclusion of animal cases could undermine the idea that the concept needs to be relativised to the interests of particular communities or perhaps needs to be split to have one concept for humans and one for non-human animals. Yet I am doubtful that encompassing animals in the extension of health or disease would serve to rule out all versions of naturalism or normativism. What remains in such a scenario will most likely be some kind of hybrid account of disease, requiring biological dysfunction but with some room for social considerations—for example, many people would not consider conditions such as post-vasectomy sterility to count as a disease. If there is a large degree of unity among the public, such an approach may also allow for a bridging of naturalism and normativism by investigating ‘(i) which dimension(s) weigh more and (ii) whether and how different dimensions are interacting’ [46, p. 266]. Indeed, I view this investigation of conflicting intuitions as the most interesting avenue for future empirical work in the philosophy of medicine. Having discussed the potential outcomes of experimental philosophy of medicine, I will now situate them in their broader context and conclude the discussion.

## Discussion

A core motivation of experimental philosophy is to shift the philosophical community’s perception of philosophy itself—moving away from the methodological idea that philosophy is about a specific set of tools and towards a more content-based conception of philosophy as a discipline that is interested in more abstract and conceptual questions. In this paper, I have argued that participants in what is perhaps the oldest debate within the field—namely, the question of how to understand health, pathology, and disease—are mistaken in thinking that a solution can be reached using conceptual analysis alone. Indeed, it is doubtful that many philosophers are actually interested in mere intuition play (despite statements to the contrary). When in the course of conducting a descriptive analysis of concepts, philosophers proceed to engage in what Lemoine calls extensional stipulation—namely, the inclusion of ‘cases of consensually healthy conditions’ (e.g., pregnancy) or the exclusion of ‘cases of consensual diseases’ (e.g., cancer) from the set of diseases [15, p. 318]—they are not purely using conceptual analysis; they are doing something else.

Fields such as bioethics and medical ethics, with strong historical ties to the philosophy of medicine, have become increasingly empirical over the years, embracing the use of surveys, interviews, and other scientific tools to improve their investigations. Philosophers of medicine would do well to follow suit. While I have limited my discussion to surveys here—given that this method is most directly associated with experimental philosophy and has straightforward application to the concepts of health and disease in the philosophy of medicine—surveys are by no means the only possible empirical tool philosophers of medicine could import. Edouard Machery proposes that experimental philosophers might also avail themselves of a diverse range of cliometric and bibliometric techniques (e.g., machine learning and topic modelling) [6]. A more thorough overview of how different empirical tools could be applied within the philosophy of medicine is offered in the introduction to this special issue [74]. Much of recent philosophy of medicine is closer to the ‘philosophy of science in practice’ and ‘philosophy *in* science’ movements, which seek to be more naturalist, more pragmatic, and fundamentally closer to science than traditional armchair investigations [75, 76]. Questions such as how medical practitioners see, use, and evaluate concepts like health, pathology, and disease are important to the philosophy of medicine. Yet these questions cannot be answered through introspection alone. They require investigative empirical methods. Even something as scientifically contested as anecdotes would offer better evidence than the mere intuitions of a single philosopher [77].

It is nevertheless important not to demand too much from experimental philosophy [78]. Conceptual analysis must proceed from shared paradigm cases in order to bring forth resolution. The more shared paradigm cases there are (to which x-phi may add), the further the discussion can proceed with conceptual analysis. There is plenty of space for improvement short of complete resolution of a philosophical debate. The latter is rare, so it would be overdemanding to expect x-phi to resolve major disputes entirely—and overblown to criticise it for failing to do so. An absence of resolution is not the same as a lack of progress.

One should therefore not expect empirical methods to produce a definitive and unique answer to the question of whether normativism or naturalism is right about the concepts of health and disease. Experimental philosophy may very well call into question the foundation of the debate itself by empirically investigating the intuitions that have given rise to it. The conflict might be settled in favour of one side, pluralism, or unification. I do not commit myself to any one of these pathways precisely because the correct course is not just a conceptual matter, but an empirical one too. That philosophers already possess all the empirical knowledge required for engaging in conceptual debates like the one about health and disease is a fantasy that needs to be abandoned. I can only hope that others will take up this baton in furtherance of an empirical turn in the philosophy of medicine.

## Footnotes

1 Despite the existence of these two camps, one should not think of either as particularly unified. The positions of normativists in particular share very little family resemblance aside from their unified dismissal of naturalistic approaches to health and disease. These anti-naturalists reject Dennett’s dictum of ‘Darwin’s universal acid’ that natural selection leaves none of our folk concepts unchanged [17, 18].

2 This assumption is not exclusive to the philosophy of medicine. It is very much conceived as the modus operandi in many conceptual debates in philosophy—indeed the general practice of philosophy itself—and has thus received an array of criticisms (see [24] for an overview).

3 Lemoine’s analysis of conceptual analysis is based on the work of Christopher Boorse [20–22], Lennart Nordenfelt [31], and Jerome Wakefield [23, 32].

4 It may be similarly unsatisfying to policymakers, funding agencies, and insurance companies faced with the line-drawing problem of which conditions should be called pathological. Biologists, on the other hand, should be much happier to embrace such vagueness, since the biological world does not lend itself to sharp distinctions. Naturalist accounts of health and disease may thus permit a higher degree of indeterminacy than normativist ones.

5 This is not to deny that conceptual analysis may have contributed positively to the conceptualisation of health and disease—progress that some will surely use to argue for a more optimistic stance on whether conceptual analysis can solve the debate. But this further step should be avoided. For additional reasons pertaining to problems with conceptual analysis that I do not have space to detail here, I am rejecting this position; see [33] for various philosophical attacks on conceptual analysis. My primary goal in this piece is merely to encourage philosophers of medicine to embrace a wider toolkit of methods in order to settle the questions they are interested in.

6 While Lemoine intends to solve this debate by motivating a naturalist account of health and disease that actually draws on the full breath of biomedical science, I offer an alternative solution here. Nevertheless, in a conference report, Lemoine et al. tentatively suggest that experimental philosophy could fruitfully be applied to the philosophy of medicine [34]. In my dissertation, in turn, I follow Lemoine’s call for an attempt to naturalise the concepts of health and disease [35].

7 For more detail on the second of these options, see my article [40], parts of which are revised here.

8 An anonymous reviewer objected that the use of scientific methods might favour, rather than negatively affect, the normativist position. For example, if one can empirically show that there are no necessary and sufficient conditions for a given state to be classified as a disease, then the normativist, rather that the naturalist, claim is validated. There are two replies to this. First, the worry is that naturalist methods favour naturalist conclusions. If the opposite were true—that naturalist methods favour normativist conclusions—there would not be much of a problem: naturalists should then happily endorse normativism for it follows from naturalist reasoning. Second, it is not true that a failure of science to provide necessary and sufficient conditions for disease implies a failure for naturalism. The biological world simply does not lend itself to basic definitions in terms of necessary and sufficient conditions, so the concept of disease may very well have to be amended if it fails to map onto its counterpart in the natural world.

9 They show this difference to be especially pronounced between the social and natural sciences, thus making scientific integration difficult.

10 From a class of his at Carnegie Mellon University.

11 Animal welfare has historically been understood in this narrow way as physiological health or well-functioning [41–43].

12 Nonetheless, what might be assumed to be a necessary requirement of experimental design has not stopped publication of various experimental philosophy papers with astonishingly small sample sizes [44].

13 See Nagatsu [45] for a longer description and analysis.

14 Note that Georges Canguilhem did not hold that animal diseases are a mere social construction and I have highlighted the importance of a phenomenological view of animal health and suffering elsewhere [49]. To tie the concept of health to the notion of subjectivity, however, might lead us to restrict these concepts to all and only those animals deemed conscious, thus transforming the debate into an even more perplexing one about the boundaries of animal sentience [50–52].

15 I have argued for this with respect to models of empirical phenomena more generally [61–66]. It might not be possible to capture notions such as ‘autism’ [67, 68] and ‘welfare’ [66, 69] within a single concept or model, since the underlying phenomena are too disunified, attempting to map onto both normative and natural features of the world. The same may hold for health and disease.

16 I have reservations against Wakefield’s view because his account makes it hard to discuss trade-offs of health and well-being that are clearly important in all kinds of bioethical debates, from those as straightforward as the potential injuries caused in mixed martial arts [71] to those as controversial as de-extinction [72]. Many debates in animal ethics are likewise centred around the question of whether it matters that we injure and kill other animals if they cannot feel pain [73]. Health and well-being can come apart.
